# Field Efficacy of *Cordyceps javanica*, White Oil and Spinetoram for the Management of the Asian Citrus Psyllid, *Diaphorina citri*

**DOI:** 10.3390/insects12090824

**Published:** 2021-09-14

**Authors:** Pasco B. Avery, Emily B. Duren, Jawwad A. Qureshi, Robert C. Adair, Matthew M. Adair, Ronald D. Cave

**Affiliations:** 1 Indian River Research and Education Center, Department of Entomology and Nematology, University of Florida, 2199 South Rock Road, Fort Pierce, FL 34945, USA; emilyduren94@gmail.com (E.B.D.); rdcave@ufl.edu (R.D.C.); 2 Southwest Florida Research and Education Center, Department of Entomology and Nematology, University of Florida, 2685 State Road 29 N, Immokalee, FL 34142, USA; jawwadq@ufl.edu; 3 The Florida Research Center for Agricultural Sustainability, 7055 33rd Street, Vero Beach, FL 32966, USA; Bob@FlaResearch.com (R.C.A.J.); mattadair3@hotmail.com (M.M.A.)

**Keywords:** citrus greening, entomopathogenic fungus, lady beetles, horticultural oils, *Isaria fumosorosea*, insecticide, natural enemies

## Abstract

**Simple Summary:**

Citrus greening is devastating the citrus industry in Florida, and conventional synthetic pesticide applications are rapidly becoming unsustainable for the control of the vector, the Asian citrus psyllid (AsCP), *Diaphorina citri*. Laboratory experiments indicate that the entomopathogenic fungus *Cordyceps javanica*, alone and in combination with horticultural oils, may offer a more sustainable strategy to manage AsCP. The field study in this paper indicated that *C. javanica* alone, *C. javanica* mixed with white oil, and spinetoram (the chemical standard) mixed with white oil significantly suppressed AsCP populations by 60–90% and 61–83% 7 and 14 days after treatment, respectively, in 2018, compared to white oil only and the untreated control treatments. Only spinetoram + oil suppressed AsCP 100% up to 7 days after treatment in 2019. AsCP’s natural enemies, mostly lady beetles, were observed on fungal-treated citrus trees and the untreated control. Overall, *C. javanica* was as effective in suppressing AsCP adults as spinetoram for up to 14 days in field conditions and was compatible with the psyllids’ natural enemies. The use of *C. javanica* in citrus-integrated pest management is suggested.

**Abstract:**

Citrus greening disease is devastating the citrus industry in Florida, and the conventional synthetic pesticide applications used to control the vector, the Asian citrus psyllid (AsCP), *Diaphorina citri*, are rapidly becoming unsustainable. Various laboratory experiments indicate that the entomopathogenic fungus *Cordyceps javanica*, alone and in combination with horticultural oils, may offer a more sustainable strategy for the management of AsCP. Field studies conducted in 2018 and 2019 in mature citrus indicated that *C. javanica* alone, *C. javanica* mixed with white oil, and the chemical standard spinetoram mixed with white oil significantly suppressed AsCP adult populations by 61–83% up to 14 days after treatment in 2018, although colony-forming units of *C. javanica* were still present on the leaves 21 days after treatment (DAT). Only spinetoram + oil significantly suppressed AsCP, by 100%, up to 7 DAT in 2019. Natural enemies of AsCP, including lady beetles, lacewing larvae and the parasitoid *Tamarixia radiata*, were observed in the fungal treatments and the untreated control. The AsCP suppression by *C. javanica* and its compatibility with beneficial organisms suggest the potential use of this entomopathogenic fungus in citrus-integrated pest management.

## 1. Introduction

The Asian citrus psyllid (AsCP), *Diaphorina citri* Kuwayama (Hemiptera: Liviidae), is a major insect pest on citrus worldwide [1]. Direct damage by AsCP is caused by injecting its stylets into the phloem to suck the sap of a citrus plant. While feeding, the AsCP can then transmit a bacterium, *Candidatus* Liberbacter asiaticus (*C*Las), that is associated with citrus greening disease, also known as huanglongbing [2,3,4,5]. At this point in time, there is no cure for HLB, and it had a major negative impact on the citrus industry [6].

To date, the primary focus for the management of greening disease has been to control the psyllid vector of the pathogen by using broad-spectrum insecticides, such spinetoram from the spinosyn chemistry class [7,8,9]. However, psyllids can escape contact with the sprays in abandoned groves or an urban environment where citrus trees are rarely treated [10]. Furthermore, due to the continual use of these various insecticides, the AsCP has developed resistance to specific chemical groups and modes of action [11]. Additionally, the use of the broad-spectrum insecticides is negatively affecting the beneficial organisms present in the citrus grove ecosystem [12,13,14], whether residential or released for the biological control of several pests. Synthetic chemicals also affect worker safety and alter the behavior and intention of the consumer to purchase organically grown produce [15,16,17]. Therefore, it is imperative to develop an integrated and sustainable management strategy for the vector to mitigate the transmission of *C*Las in this pathosystem [5]).

An effective management strategy can incorporate an entomopathogenic fungus endemic to the area where it is compatible with beneficial organisms. In 2008, Meyer et al. [18] found AsCP adults infected with an entomopathogenic fungus, identified as *Isaria fumosorosea* Wize, on citrus leaves in Polk County, Florida. Avery et al. [19,20] tested strains of the fungus, including the Apopka strain originally isolated from whiteflies in Florida [21] and contained in the formulated product PFR-97™ 20% WDG (Certis USA LLC, Columbia, MD, USA), against AsCP adults to determine whether they were susceptible to infection under laboratory conditions. In 2017, the taxonomy of this fungus was updated to *Cordyceps fumosorosea* (Wize) Kepler, B. Shrestha and Spatafora (Hypocreales: Cordycipitaceae). The species identification, based only on the morphological characteristics, was subsequently corrected with molecular studies (Cabanillas et al. [22] and Dunlap et al. [23]), which indicated that it should be *C. javanica* (Frieder. and Bally) Kepler, B. Shrestha and Spatafora, 2017 [24], abbreviated hereafter as *Cja*.

Leaf disk bioassays indicated that AsCP adults are susceptible to infection by the Apopka strain of *Cja*; upon infection, there is a sudden reduction and eventual cessation of feeding over time [20]. Subsequent laboratory and greenhouse studies demonstrated that the *Cja* Apopka strain is compatible with various agrochemicals used for the management of AsCP and other insect pests in Florida citrus groves [25,26,27]. When blastospores of the *Cja* Apopka strain were mixed with citrus or petroleum oils in a suspension and sprayed on leaves exposed afterwards for AsCP adults, the mean survival rate of the insects was significantly lower compared to the fungal suspension applied alone [28]. *Cordyceps javanica* is also compatible with various beneficial organisms [29,30], including lady beetles [31,32,33] and the parasitoid *Tamarixia radiata* (Waterson) (Hymenoptera: Eulophidae), which are natural enemies of AsCP [13,34,35,36,37,38].

Although the Apopka *Cja* Apopka strain is compatible and efficacious in the laboratory and greenhouse with oils and beneficial insects, it needs to be tested under field conditions. The objective of our study was to determine the efficacy and persistence of the *Cja* Apopka strain alone or mixed with white oil under citrus grove conditions compared with spinetoram, a chemical insecticide standard commonly used for the management of adult AsCP [7]. In addition, the effects of the treatments on the presence of natural enemies of AsCP were assessed.

## 2. Materials and Methods

### 2.1. Study Site and Experimental Treatments

The field trials were conducted at the Florida Research Center for Agricultural Sustainability, Vero Beach, Indian River County, FL, in two rows of ‘Minneola’ Tangelos grafted onto Kinkoji rootstock. The trees were planted on 8 October 2009 with a 0.30 m × 0.64 m tree spacing (358 trees per ha). The trees were symptomatic of greening disease in relatively good health and producing fruit. During the study in 2018 and 2019, the daily mean temperature (°C), mean relative humidity (%) and precipitation (cm) parameters were obtained from the Weather Underground Network at https://www.wunderground.com/weather/us/fl/vero-beach/ (accessed on 15 June 2020).

There were five trees per linear block and four replicated blocks arranged in a randomized complete block design per treatment. Two unsprayed buffer trees separated the blocks. Each block received one of the following five treatments: (1) untreated control; (2) white oil (JMS Stylet-Oil^®^, JMS Flower Farms, Inc., Vero Beach, FL, USA); (3) the *Cja* Apopka strain (PFR-97™ 20% WDG, Certis USA LLC, Columbia, MD, USA) mixed with water (~10^6^ blastospores/mL); (4) the *Cja* Apopka strain mixed with water and white oil; (5) spinetoram (Delegate WG™, Dow AgroSciences LLC, Indianapolis, IN, USA) with white oil added. The trees did not receive any insecticide applications for 6–8 weeks prior to the application of the treatments, but they were fertilized according to commercial standards and kept weed-free with herbicide applications according to the University of Florida Citrus Pest Management Guide [39].

Into a clean plastic pail, 907 g PFR-97 were added to 19 L dechlorinated (Prime^®^ Dechlorinator, Seachem Laboratories, Madison, GA, USA) potable water and mixed with a clean paint mixer attached to a battery-operated drill. The suspension was allowed one hour for the blastospores to imbibe prior to spraying. The suspension was added to 227 L dechlorinated water, and the mixture was poured into a calibrated, stainless steel mixer equipped with an electric-powered mixing propeller. The resulting 246 L spray suspension was pumped directly into the sprayer. The blastospores of *Cja* in the PFR-97 product prior to each application were determined to be 100% viable using the technique described by Kumar et al. [28]. The white oil alone and the spinetoram (283.5 g/ha rate) + oil treatments were mixed directly in the sprayer with 246 L of water that were measured and pumped into the sprayer by means of the same stainless steel mixer. For the oil only, *Cja* + oil, and spinetoram + oil treatments, 1% *v*/*v* of JMS Stylet-Oil was added last to the sprayer. The spray tank was thoroughly cleaned after each application.

All treatments were applied at dusk with a commercial air blast-sprayer (Rears Power Blast Model PB-500) at a ground speed of 2.4 kmph and a pump pressure of 1310 kPa. In Trial 1, a single application on 2 May 2018 to the west or east side of the trees used five ceramic Disc-Core type hollow cone spray tips (#5 Disc, #45 Core, TeeJet^®^, Wheaton, IL, USA) calibrated to deliver 608 L/ha spray material [40]. In Trial 2, a single application on 17 April 2019 to the west and east sides of all trees used seven Conjet air induction hollow cone spray tips (AITX 8004VK, TeeJet^®^, Wheaton, IL, USA) calibrated to deliver 1525 L/ha of spray material [40].

### 2.2. Spore Deposition

Prior to the spray applications of *Cja* in Treatments 3 and 4, plastic coverslips (Fisherbrand^®^ 22 mm × 22 mm, Fisher Scientific, Pittsburgh, PA, USA) were pinned to the abaxial and adaxial sides of randomly chosen leaves in order to determine blastospore deposition per mm^2^, as described by Avery et al. [33]. The coverslips were allowed to air dry overnight post-application (~12 h). After removal the next day, the coverslips were pinned to a styrofoam cooler lid and transported to the Entomopathogenic Fungi Research Laboratory at the Indian River Research and Education Center in Fort Pierce, FL. They were examined by the technique described by Avery et al. [33]. However, a Leica DM500 stereo compound light microscope (400×) with a built-in camera (Leica Microsystems Inc., Buffalo Grove, IL, USA) was used, and the blastospores were counted in each area viewed on the videoscreen.

### 2.3. AsCP and Natural Enemy Abundance

The scouting for adult AsCP and natural enemies was conducted weekly by using the stem tap sampling method [34] prior to and after the spray applications. The four cardinal directions of each tree were sampled [41]. Four stem tap samples were taken per tree, weekly, for 3 weeks (Trial 1) and 4 weeks (Trial 2). A total of 64 stem tap samples were taken from 16 randomly chosen trees (4 trees per block) per treatment per week. The AsCP adults, lady beetles, and other natural enemies per stem tap sample were recorded.

### 2.4. Cordyceps javanica *Field Persistence and Pathogenicity*

The field persistence and viability of *Cja* fungal propagules on the citrus leaves over time were assessed using the leaf disk bioassay technique described by Avery et al. [33]. In brief, 5 (Trial 1) and 8 (Trial 2) randomly chosen leaves selected from all of the trees in each block of the two fungal treatments and the untreated control were detached 3 days before the treatment applications, and 1, 7 and 14 days after the treatments (DAT). The leaves were placed in resealable plastic bags, stored in a styrofoam cooler with an ice pack, and transported to the laboratory. The samples were immediately transferred from the cooler to a refrigerator at 4 °C until they were assessed.

From each detached leaf, three disks were removed with cork-borers. One large (25 mm diam) disk was removed from the center of the leaf with the midrib, and 2 small (10 mm diam) disks were removed from each side of the midrib. The large disk was used to determine the AsCP median and mean survival times (days) and mortality (%) over time. The two smaller disks were used to determine the viability of the propagules (blastospores, conidia, and/or hyphae).

To determine AsCP median survival time (ST_50_) and mean survival time, the large leaf disks were individually placed on water agar in a Petri dish (35 mm × 10 mm), as described by Hall and Nguyen [14], but the leaves in the field were sprayed on both sides, the disk was not imbedded in the agar but placed on top of the solidified agar. The AsCP adults were obtained from a colony at the USDA-ARS Horticultural Research Laboratory in Fort Pierce, Florida, and were reared as described by Avery et al. [19]. Adult AsCP (<1 week old) pre-cooled at 4 °C for a few minutes were transferred to the inside surface of a Petri dish lid using a camel-hair brush. The lid was placed on the dish bottom, and the psyllid naturally transferred onto the leaf disk. The bioassay plates, each with a single psyllid, were sealed with Parafilm^®^ M (Bemis Co, Inc., Neenah, WI, USA). The plates were stacked in threes (one from each treatment), and the stacks were randomly ordered on cafeteria trays that were placed inside an environmental chamber held at 25 °C with a 16 h photophase for 24 h. After this time, the Parafilm was removed from the dishes, and the dead AsCP were counted; the dead individuals were laying on their side or did not move when gently prodded. Only the dishes with live AsCP were placed back on the cafeteria tray and returned to the growth chamber for continued daily observation for 8 days. The dead psyllids were removed, placed in individual Petri dishes (35 mm × 10 mm) with moistened filter paper, sealed with Parafilm, and returned to the growth chamber. These sealed dishes were incubated ~7 days to confirm mycosis based on the *Cja* phenotype.

The small leaf disks were placed in a 15 mL centrifuge tube (Fisherbrand™; ThermoFisher Scientific, Waltham, MA, USA) containing 3 mL 0.01% Triton X-100 (Trial 1) or 2 mL 0.01% Tween 80 (Trial 2), and were vortexed for 15 s. The propagules in the suspensions were allowed at least 5 min to settle to the bottom of the tube. A 100 μL aliquot of each suspension was removed from the bottom of the tube and randomly dribbled onto Petri dish (100 mm × 15 mm) plates containing a selective potato dextrose agar (PDA) solid medium with two bactericides (streptomycin and chloramphenicol at 0.1%) and 0.5% dodine (Dodin Pestanal: Sigma Aldrich, Inc., Saint Louis, MO, USA) (PDA-dodine). The suspension on the plates was spread with a flame-sterilized glass spreader. The Petri dishes were sealed with Parafilm, randomized on cafeteria trays as described above, transferred to the environmental chamber, and incubated under the same conditions described above. The colony-forming units (CFUs) with the *Cja* phenotype growing on the agar plates were counted 2 weeks later.

### 2.5. Statistical Analyses

The treatment means were compared with a one-way ANOVA and separated *post hoc* where appropriate with LSD or Tukey’s HSD test (*α* = 0.05). All statistical analyses were conducted using SAS Proc GLM procedures and executed on a PRO WIN 6.1 platform (SAS 2002–2012; SAS Institute Inc., Cary, NC, USA). The ST_50_ of the adult AsCP per treatment were compared with Kaplan-Meier survival analysis, followed by a log rank test (SAS JMP 8 for Windows 2013). The mean AsCP survival times per treatment were compared with the untreated control using the LSD test (*α* = 0.05).

## 3. Results

### 3.1. Trial 1—2018

#### 3.1.1. Weather Parameters

**Figure 1:**
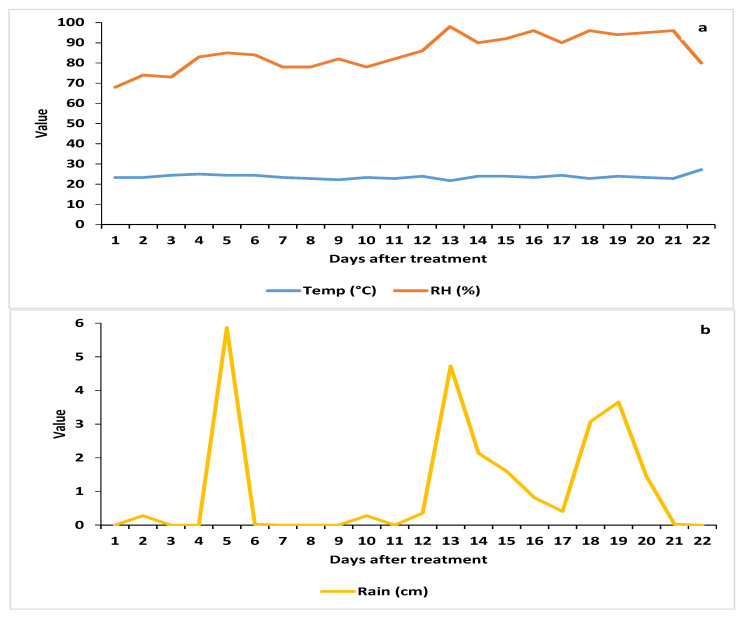
Daily weather parameters during the study in 2018: (**a**) mean temperature and relative humidity; (**b**) precipitation.

The daily weather parameters (mean ± SE) for 3 weeks after the treatment applications (Figure 1) were as follows: temperature, 23.7 ± 0.2 °C (range: 19.3–28.2 °C); relative humidity, 85.4 ± 1.8% (67.8–98.5%); precipitation (rain), 1.1 ± 0.4 cm (0–5.9 cm). The cumulative rainfall was 0.28, 6.17, 9.09, and 9.42 cm at 1, 7, 14 and 21 DAT, respectively. The total weekly precipitation over the 21 days averaged 8.2 cm.

#### 3.1.2. Spore Deposition

The mean number of deposited blastospores per mm^2^ (±SE) on the citrus trees sprayed with *Cja* alone was significantly higher (*t*_58_ = −2.33, *p* = 0.0234) on the adaxial side (845.8 ± 182.1) than on the abaxial (392.1 ± 69.4). On the trees sprayed with *Cja* + oil, the mean number of deposited blastospores per mm^2^ (±SE) was significantly higher (*t*_48_ = −2.18, *p* = 0.0342) on the adaxial side (687.1 ± 147.8) than on the abaxial (280.0 ± 53.0). There was no significant difference between the *Cja* alone and *Cja* + oil treatments in the mean number of blastospores per mm^2^ deposited on the adaxial (*t*_58_ = 0.68, *p* = 0.5012) or the abaxial (*t*_48_ = 1.17, *p* = 0.2464) side of the leaves.

#### 3.1.3. AsCP and Natural Enemy Abundance

**Figure 2:**
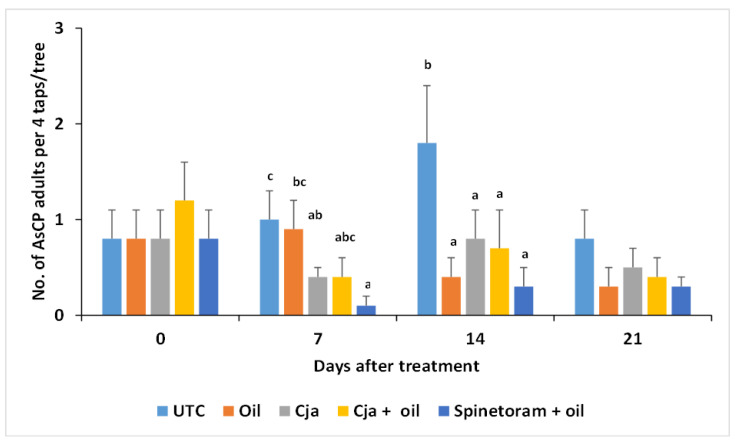
Mean number ± SE of Asian citrus psyllid (AsCP) adults per four stem taps per tree for each treatment on different days after treatment in Trial 1. The mean values with a different letter above the ±SE bar are significantly different days after treatment (LSD test; *p* < 0.05). There were no significant differences among the treatments (*p* > 0.05) at 0 and 21 days after the treatment. The untransformed data are presented. UTC = untreated control; Cja = *Cordyceps javanica*.

The counts of AsCP adults per four stem taps per tree were not significantly different among the treatments prior to the treatment applications (baseline: *F*_4,60_ = 0.41, *p* = 0.9840). *Cordyceps javanica*, applied either alone or mixed with oil, had the same effectiveness for the suppression of AsCP 7 days after treatment as spinetoram + oil (Figure 2), and all three provided a significant reduction compared to the untreated control (*F*_4,60_ = 3.63, *p* = 0.0103). All product treatments were more effective in suppressing AsCP 14 DAT compared to the untreated control (*F*_4,60_ = 3.06, *p* = 0.0232). By 21 DAT, none of the treatments were significantly (*p* > 0.05) better at suppressing the psyllid population compared to the untreated control.

Four species of AsCP natural enemies were encountered in the stem tap samples (Table 1). Three species were lady beetles: the multicolored Asian lady beetle, *Harmonia axyridis* (Pallas); the metallic blue lady beetle, *Curinus coeruleus* (Mulsant); and the blood red lady beetle, *Cycloneda sanguinea* (Linnaeus). At 0 DAT, no natural enemies were observed in any of the treatment plots.

#### 3.1.4. *Cordyceps javanica* Field Persistence and Pathogenicity

**Figure 3:**
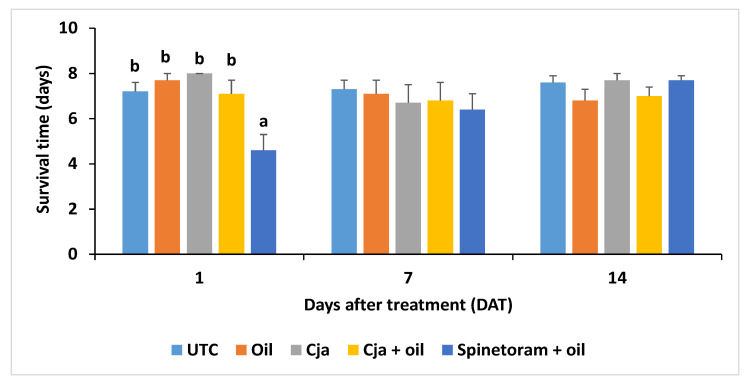
Mean survival time ± SE of AsCP adults on disks from leaves collected at 1, 7 and 14 days after treatment in Trial 1. The mean values with a different letter above the ±SE bar were significantly different at 1 day after treatment (Tukey’s test; *p* < 0.05). The mean survival times were not significantly different (*p* > 0.05) among the treatments at 7 and 14 days after treatment. The untransformed data are presented. UTC = untreated control; Cja = *Cordyceps javanica*.

The ST_50_ of adult AsCP was significantly different (χ^2^ = 28.7, df = 4, *p* < 0.0001) among the treatments at 1 DAT only. The median survival times ± SE of adult psyllids on disks from leaves collected 1 DAT from the untreated control, oil only, *Cja* alone, *Cja* + oil, and spinetoram + oil treatments were 4.9 ± 0.1, 4.0 ± 0.0, 7.0 ± 0.0, 3.8 ± 0.3, and 3.7 ± 0.3 after 7 days, respectively. At 3 days before the treatment and 7 and 14 DAT, the ST_50s_ were not significantly (*p* > 0.05) different compared to the untreated control. At 1 DAT, the mean survival time of AsCP on the leaves sprayed with spinetoram + oil was significantly less compared to the other treatments (*F*_4,44_ = 6.33, *p* = 0.0004), which were all similar (Figure 3). At 3 days before the treatment and 7 and 14 DAT, the survival time of the AsCP did not differ among the treatments (*p* > 0.05).

The mean numbers of CFUs per dish recovered from leaves collected 1, 7, 14 and 21 DAT in the treatment with *Cja* only were 0, 0, 0, and 10, respectively. For the treatment with *Cja* + oil, the mean numbers of CFUs per dish recovered from leaves collected 1, 7, 14, and 21 DAT were 0, 80, 0 and 20, respectively. No CFUs were found in the untreated control throughout the trial.

### 3.2. Trial 2—2019

#### 3.2.1. Weather Parameters

**Figure 4:**
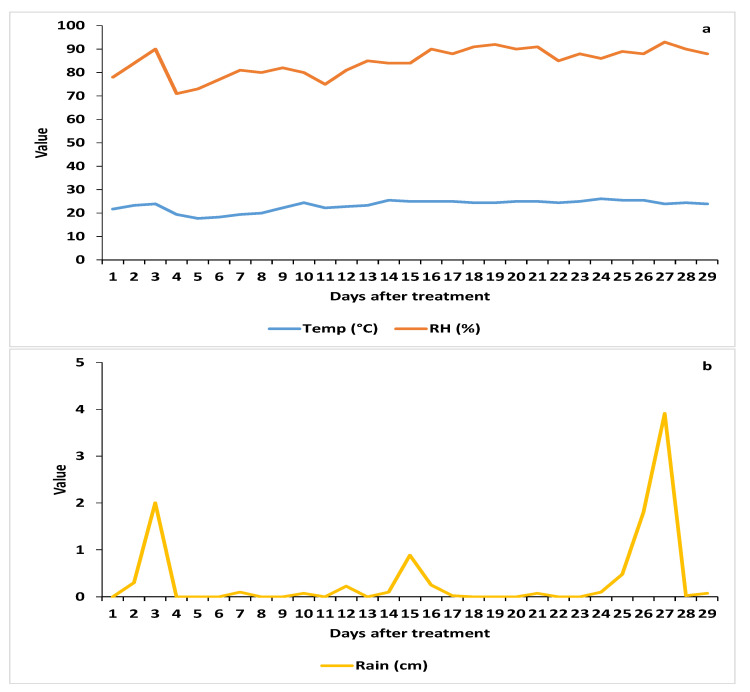
Daily weather parameters during the study in 2019: (**a**) mean temperature and relative humidity; (**b**) precipitation.

The daily weather parameters (mean ± SE) for 4 weeks after the treatment applications (Figure 4) were as follows: temperature, 23.3 ± 0.4 °C (range: 17.4–29.9 °C); relative humidity, 84.6 ± 1.1% (71–93%); precipitation (rain), 0.4 ± cm (0–3.9 cm). The total weekly precipitation averaged 2.6 cm. The cumulative rainfall at 1, 7, 14, 21 and 28 DAT was 0.3, 2.7, 4.0, 4.4 and 10.7, respectively.

#### 3.2.2. Spore Deposition

The mean number of blastospores per mm^2^ (±SE) deposited on citrus trees sprayed with *Cja* alone was similar (*t*_78_ = −1.08, *p* = 0.2840) on the adaxial (598.4 ± 52.5) and abaxial (520.0 ± 50.2) sides of the leaves. On trees sprayed with *Cja* + oil, the mean number of deposited blastospores mm^2^ (±SE) was also similar (*t*_78_ = −0.53, *p* = 0.5947) on the adaxial (733.4 ± 61.5) and abaxial (280.0 ± 53.0) sides. There was no significant difference between the *Cja* alone and the *Cja* + oil treatments in the mean number of blastospores mm^2^ deposited on the adaxial (*t*_78_ = −1.67, *p* = 0.0990) or abaxial (*t*_78_ = −1.84, *p* = 0.0702) side of the leaves.

#### 3.2.3. AsCP and Natural Enemy Abundance

**Figure 5:**
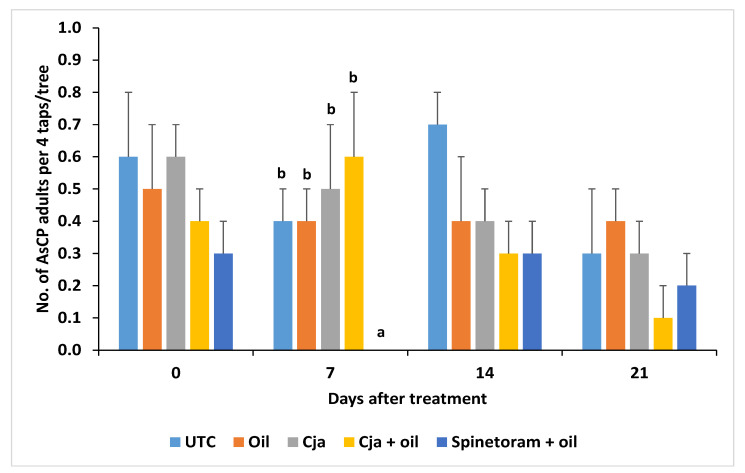
Mean number of AsCP adults per four taps per tree for each treatment at different days after treatment in Trial 2. The mean values with a different letter above the ±SE bar are significant for each day after treatment (LSD test; *p* < 0.05). There were no significant differences among the treatments (*p* > 0.05) at 0, 14 and 21 days after treatment. The untransformed data are presented. UTC = untreated control; Cja = *Cordyceps javanica*.

Spinetoram + oil was more effective (*F*_4.60_ = 3.02, *p* = 0.0246) in suppressing the psyllid population than the other treatments at 7 DAT (Figure 5). After this time, there were no significant differences in the number of AsCP (*p* > 0.05).

Five species of AsCP natural enemies were encountered in the stem tap samples (Table 2). The trees sprayed with *Cja* and those in the untreated control had the highest total number of natural enemies. Lacewing larvae and adult and larval *C. coeruleus* were observed only in the *Cja* alone and the non-sprayed control treatments. Larval and adult *C. sanguinea* were encountered in all treatments.

#### 3.2.4. *Cordyceps javanica* Field Persistence and Pathogenicity

**Figure 6:**
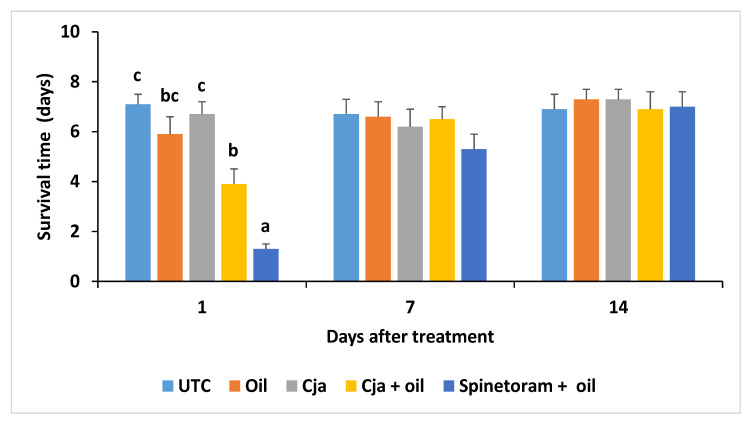
Mean survival time ± SE of AsCP adults on disks from leaves collected at 1, 7 and 14 days after treatment for Trial 2. The mean values with a different letter above the ±SEM bar are significantly different at 1 day after treatment (Tukey’s test; *p* < 0.05). The mean survival times were not significantly different (*p* > 0.05) among the treatments at 7 and 14 days after treatment. The untransformed data are presented. UTC = untreated control; Cja = *Cordyceps javanica*.

The ST_50_ for adult AsCP on leaves collected at 1 DAT were significantly different (χ^2^ = 53.2, df = 4, *p* < 0.0001) among the treatments. The median survival times ± SE of the adult psyllids on disks from leaves collected 1 DAT from the untreated control, oil only, *Cja* alone, *Cja* + oil, spinetoram + oil treatments were 6.6 ± 0.3, 4.9 ± 0.5, 6.3 ± 0.4, 3.6 ± 0.4, and 1.3 ± 0.2 after 7 days, respectively. At 3 days before treatment and 7 and 14 DAT, the ST_50_ were not significantly (*p* > 0.05) different compared to the untreated control. At 1 DAT, the mean survival time of AsCP on leaves sprayed with spinetoram + oil was significantly less (*F*_4,39_ = 22.3, *p* < 0.0001) compared to the other treatments (Figure 6). Furthermore, the AsCP mean survival time on leaves treated with *Cja* + oil was significantly less than that on leaves from the *Cja* alone treatment and the untreated control. However, the survival time of AsCP on leaves with *Cja* + oil was similar to the survival time on leaf disks with oil only. At 3 days before treatment and 7 and 14 DAT, the survival time of the AsCP did not differ among the treatments (*p* > 0.05).

The mean number of CFUs per dish recovered from the leaves collected in the treatment with *Cja* only for 1, 7, 14, 21 and 28 DAT was 112, 5, 0, 0, and 0, respectively. For the *Cja* + oil treatment, the mean number of CFUs per dish recovered from the leaves collected 1, 7, 14 and 21 DAT was 114, 1, 0, 0 and 0, respectively. No CFUs were found in the untreated control throughout the trial.

## 4. Discussion

The results from the field trial in 2018 indicate that the application of *Cja* blastospores alone and mixed with oil was as effective as applying spinetoram to the citrus trees for the suppression of AsCP populations up to 14 DAT. However, in 2019, the same results were not observed, when spinetoram was superior to all of the other treatments in suppressing the very low psyllid population up to 7 DAT. After that time, none of the treatments were effective in suppressing the AsCP. In both trials, the environmental conditions were not optimal for the growth and pathogenesis of *Cja* for the infection of AsCP adults that encountered the fungal propagules on the leaf phylloplane. The best temperature for the germination and growth of *Cja* is ≥25 °C, so the average field temperature in both trials (23–24 °C) was slightly below the optimum [42], although it did rise to 28–30 °C on some days. The blastospores of *Cja* germinate 6–8 h post-spray at 25 °C [43]. Therefore, temperatures <25 °C would slow the germination of them after the psyllids become contaminated with the residual spores on the leaf surface. The relative humidity during the trials, however, was conducive to fungal germination and growth [44]. Another environmental factor that could have affected the efficacy and CFU counts of the fungal propagules present on the leaf surface over time is solar radiation, which we did not measure in this study. Smits et al. [45] demonstrated that the exposure of *C. fumosorosea* propagules to UVB irradiation was detrimental to their germination, CFU formation, and persistence over time.

In the 2018 trial, spore deposition rates for both fungal treatments were higher on the adaxial side of the leaves compared to the abaxial side. However, these data were not congruent with the CFU counts over 21 days. The reasons for this discrepancy could be: (1) the citrus trees were sprayed on one side only; (2) the randomly sampled leaves collected for analysis from the east side of the tree canopy were either partially covered with spores, or not at all; and/or (3) the rainfall post-spray may have washed the spores off the leaves overtime.

Because the citrus tree canopies had been thinned by HLB infection, we assumed that the pressure generated by the air blast sprayer would be sufficient to penetrate through the tree canopy and cover the leaves on the east (unsprayed) side of the tree. However, the leaves collected randomly from the different sides of the trees for the bioassays could have had very few, if any, spores deposited on them from the spray application. It is also possible that, by chance, the small leaf disks removed from each side of the midrib contained few or no propagules, resulting in zero CFUs being counted on the PDA-dodine plates. In addition, the low numbers of propagules on the large disk removed from the same leaves could account for the high survival of the AsCP after exposure to the leaves in the leaf disk bioassay. The rainfall during both trials (Figure 1b and Figure 4b) would negatively affect the results of the CFU counts.

Spraying both sides of the trees, as was performed in the 2019 trial, apparently provided better coverage, because the blastospore deposition on both sides of the leaves was similar, and the CFU counts were higher. The spinetoram + oil treatment was most effective at 7 DAT, but after that time its efficacy in suppressing the adult AsCP population was no better than any of the other treatments compared to the untreated control. This loss in treatment efficacy after 7 DAT may be due to the rainfall, which could have washed the active ingredient or fungal spores off the leaves. In fact, the survival time of adult AsCP on leaves sprayed with spinetoram + oil was significantly less than that on the leaves from the other treatments only at 1 DAT. The reduction in CFUs over time for all fungal treatments indicated that the reduction in efficacy over time was likely related to the rainfall. The leaves and coverslips were collected from the field at 1 DAT when it was dry, but the rainfall started 3 DAT and continued over time, negatively affecting the field efficacy of the treatments, as it did in 2018.

In both trials, the most abundant natural enemies on the treated and untreated trees were lady beetles from four genera. All of the lady beetle species observed in this field study are known to be predators of AsCP eggs and nymphs in Florida groves [13]. Although only one *T. radiata* was seen in the tap samples, this is no indication that the parasitoid was widespread in the grove because they were being released in the grove on a regular basis. It is notable that a similar number of natural enemies were found in plots sprayed with PFR-97 to those in the untreated plots. This demonstrates that *Cja* is compatible with the natural enemies commonly present in the citrus agroecosystem. In corroboration of this finding, Avery et al. [33]) observed *C. coeruleus* foraging on ficus plants sprayed with PFR-97.

## 5. Conclusions

There have been several different methods employed for the use of *Cordyceps* spp. against AsCP in field conditions, and these include: (1) spraying the target pest on the foliage [46,47,48,49,50]; (2) releasing entomopathogenic fungi using autodisseminators [51,52]; and (3) a combination of these methods [37]. Each method employed above using fungal formulations containing *Cordyceps* spp. has been reported to be efficacious for up to 14 DAT in the field, which agrees with our findings.

The field efficacy of *Cordyceps* spp. is dependent on environmental conditions, i.e., the temperature and relative humidity. Rainfall, which will wash blastospores of *Cordyceps* spp. off the leaf surface due the hydrophilic property of the fungal spore type [53], has been noted by some authors to be one of the environmental factors to have the greatest negative influence on the persistence of propagules under field conditions. This observation also agrees with our findings when applying *Cja* blastospores. Therefore, more research is needed to find a solution to this dilemma, possibly using rain-fast adjuvant products. However, each rain-fast product must be tested to determine its compatibility with the *Cja* blastospores in vitro prior to its application in the field. Based on this study and other similar studies, in order to enhance the field efficacy and persistence of the spray application over time, the field environmental conditions and electrostatic properties of the propagules need to be considered when using entomopathogenic fungal biopesticides.

Considering that the application of spinetoram was as effective or more efficacious in suppressing the adult AsCP population in comparison to Cja + alone, perhaps its combination with the fungal treatment could be more efficacious when applied against the AsCP under field conditions. Fiaz et al. [49] demonstrated that the application of spirotetramat along with *Cja* demonstrated a significant synergistic effect for the reduction of AsCP populations. These authors recommended this combination treatment to citrus growers when spraying against sucking pests. Therefore, a combination of spinetoram and the *Cja* Apopka strain may produce a similar synergistic effect; however, this combination treatment needs to be evaluated under laboratory conditions and, if compatible, in the field. Our field study provides more supportive evidence that *Cordyceps* spp. can be used in an integrated pest management program with natural enemies to suppress AsCP populations.

## Figures and Tables

**Table 1:** Natural enemies of Asian citrus psyllid detected in the stem tap samples in Trial 1.

	Lady Beetles ^a^					Parasitoid ^a^	
Treatment	*Ha*-a	*Cc*-l	*Cc*-a	*Cs*-l	*Cs*-a	*Tr*-a	Total NE ^b^
Oil						1	1
*Cja* ^c^		1			1		2
*Cja* + oil		1			1		2
Spinetoram + oil			1				1
Untreated control	1						1

^a^*Ha* = *Harmonia axyridis*; *Cc* = *Curinus coeruleus*; *Cs* = *Cycloneda sanguinea*; *Tr* = *Tamarixia radiata*; l = larva; a = adult; ^b^ NE = natural enemies; ^c^ *Cja* = *Cordyceps javanica*.

**Table 2:** Natural enemies of the Asian citrus psyllid detected in stem tap samples in Trial 2.

	Lady Beetles ^a^						Lacewing ^a^	
Treatment	*Ha*-a	*Cc*-l	*Cc*-a	*Cs*-l	*Cs*-a	*Ov*-a	lw-l	Total NE ^b^
Oil	1				1			2
*Cja* ^c^	2		3		2	1	1	9
*Cja* + oil	2			1	1			4
Spinetoram + oil	1				2			3
Untreated control		1	1	3	5		1	8

^a^*Ha* = *Harmonia axyridis*; *Cc* = *Curinus coeruleus*; *Cs* = *Cycloneda sanguinea*; *Ov* = *Olla v-nigrum*; lw-l = unidentified lacewing larva; l = larva; a = adult; ^b^ NE = natural enemies; ^c^ *Cja* = *Cordyceps javanica.*

## Data Availability

The data presented in this study are available on request from the corresponding author.
